# Case Report of Hydatid Cyst in the Pulmonary Artery Uncommon Presentation: CT and MRI Findings

**DOI:** 10.1155/2018/1301072

**Published:** 2018-05-15

**Authors:** Arwa Almutairi, Sulaiman Al Rajhi

**Affiliations:** ^1^King Saud Bin Abdulaziz University for Health Sciences, Riyadh, Saudi Arabia; ^2^Medical Imaging Department, King Abdulaziz Medical City, Riyadh, Saudi Arabia

## Abstract

**Background:**

Hydatid cysts can be found in any organ. In adults, the liver and lungs are the most common locations; hydatid cysts in the pulmonary artery are rare.

**Clinical Case:**

We present the case of an 86-year-old female with a history of hepatic hydatid cyst since 2012, who presented with complaints of chronic productive cough, yellowish-green sputum, and dyspnea. CT and MRI showed multiseptate hydatid cysts in the right pulmonary artery.

## 1. Introduction

Hydatid disease may be caused when the tapeworm* Echinococcus* goes through its metacestode stage to form larvae. Most human infections worldwide are caused by* E. granulosus* that causes cystic echinococcosis [1]. Echinococcosis can occur in any organ as primary echinococcosis or can be found elsewhere due to spread of metacestodes from primary sites (secondary echinococcosis) [1]. In adults, the liver is the most common location for the cysts, accounting for 70% of the cases, while the lungs are the second most common location (20%); other locations include the spleen and kidneys, which account for 2% each [2]. Pulmonary artery involvement by hydatid cyst is rare. Here, we report a case of hydatid cyst located in the right pulmonary artery.

## 2. Case Report

**Figure 1:**
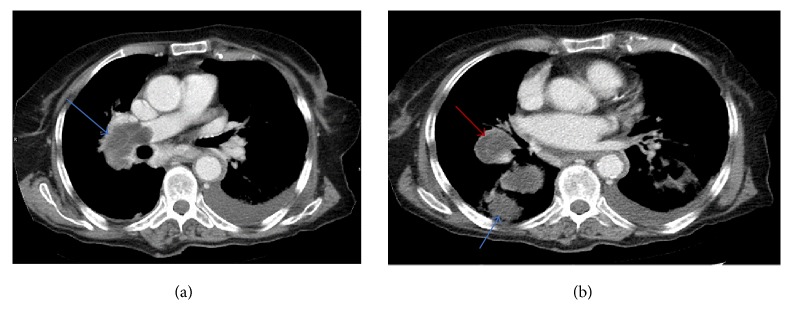
(a-b) CT axial images show pulmonary artery with multiseptate hydatid cysts (red arrows), seen in the right main pulmonary artery and the lower lobar branches of the right pulmonary artery, along with other parenchymal cysts (blue arrows).

**Figure 2:**
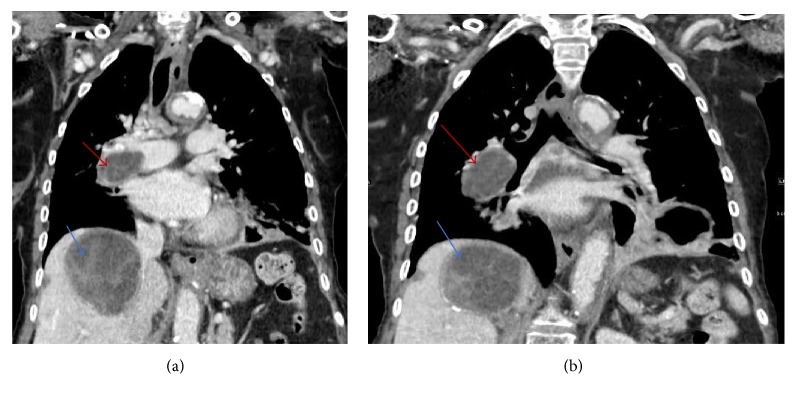
(a-b) CT coronal images show right main pulmonary artery hydatid cysts (red arrows) along with a cyst of the right hepatic lobe (blue arrows).

**Figure 3:**
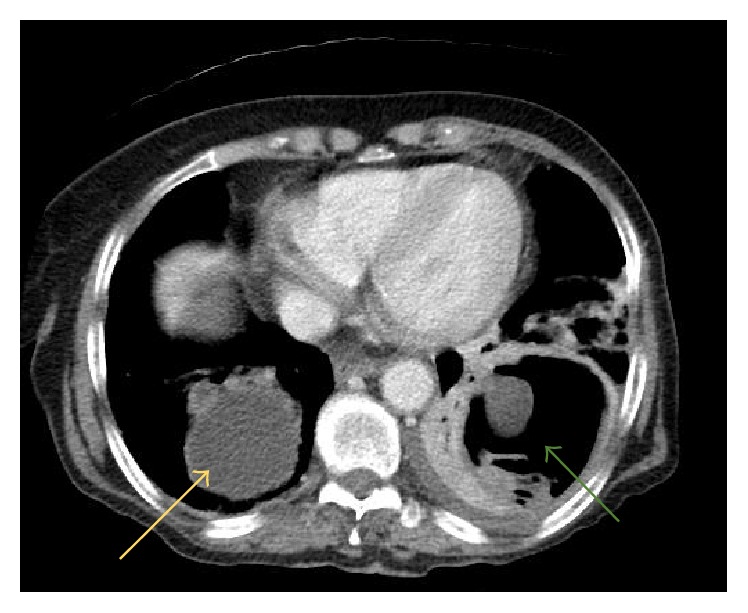
CT axial image shows a large cyst in the right lower lobe representing parenchymal hydatid cyst (yellow arrow); there is also a large cyst with air seen in the left lower lobe representing hydatid cyst in the left lung (green arrow).

**Figure 4:**
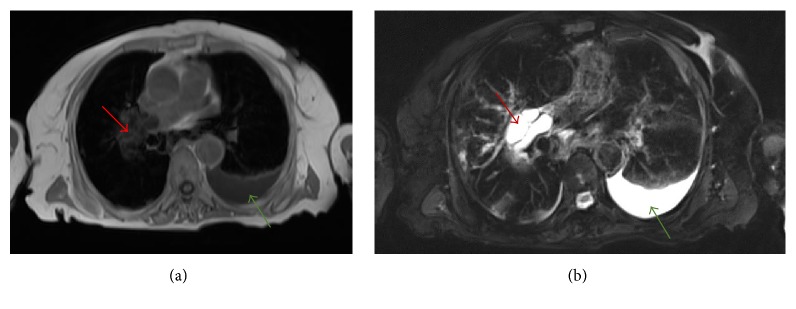
(a-b) Axial T1 and T2 weighted images show hydatid cyst (red arrows) in the right main pulmonary artery and small pleural effusions on the left side (green arrows).

**Figure 5:**
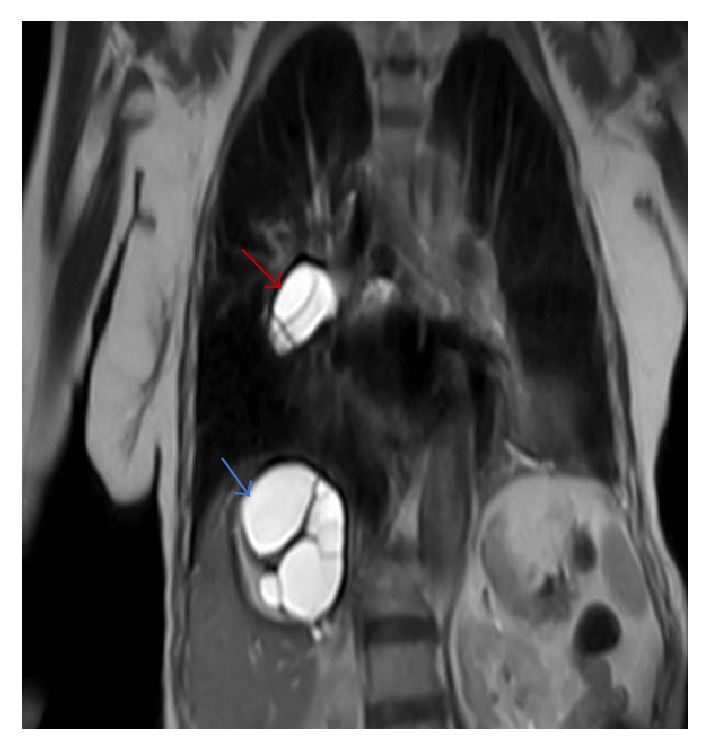
A coronal T2 weighted image shows a high signal intensity cystic lesion (red arrow) within the right main pulmonary artery with multiseptate high intensity cystic lesion on the right hepatic lobe (blue arrow).

**Figure 6:**
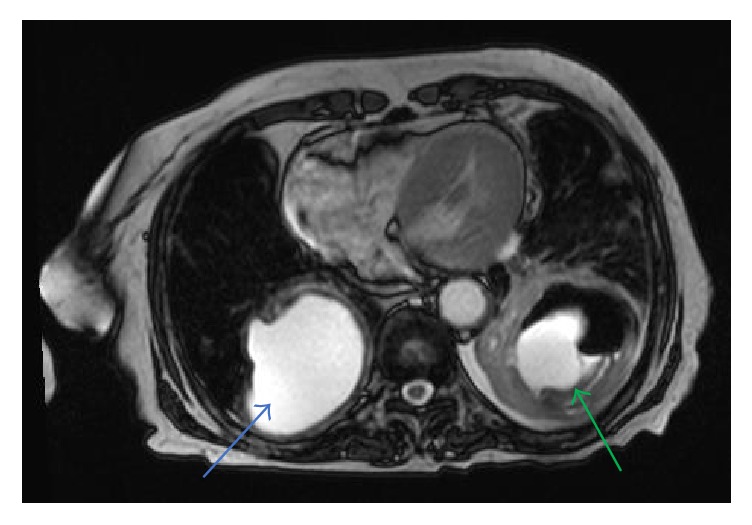
Axial T2 weighted image shows a large cyst in the right lower lobe representing parenchymal hydatid cyst (blue arrow); there is also a large cyst with air seen in the left lower lobe representing hydatid cyst in the left lung (green arrow).

An 86-year-old female patient with a history of hepatic hydatid cyst since 2012 presented in November 2016 with a history of productive cough, yellowish-green sputum with mild shortness of breath, and loss of appetite. She had no complaints of chest pain, fever, palpitations, nausea, or vomiting and did not report any change in bowel habits or urinary symptoms. The patient was from a rural area. She had a history of long-term antiparasitic medication; however, details about the indication for and type and course of therapy were not available in the medical records. On respiratory examination, the lungs were found to be clear with good air entry bilaterally; fine crepitation was noted. The patient was admitted for investigation. Sputum culture showed scanty growth of normal respiratory flora, and rhino virus was detected in nasal swab culture. Chest X-ray showed a mass-like opacity in the right lower zone, in the right paracardial area. A nonhomogenous airspace opacity was observed in the left lower lung zone associated with a small left pleural effusion that indicated atelectasis or infection as differential diagnosis. Thoracic computed tomography (CT) was performed to investigate the mass-like opacity in the right lower zone. The CT showed multiple intraluminal cystic filling defects in the right main pulmonary artery and right lobar branches, mostly pulmonary artery hydatid cysts (see Figures 1 and 2). There was one large cyst in the right lower lung lobe representing a parenchymal hydatid cyst and another large cyst existed with air in the left lower lobe representing hydatid cyst in the left lung (see Figure 3). There was minimal bilateral pleural effusion, more on the left side. We conducted magnetic resonance imaging (MRI) of the thorax, which confirmed the presence of the cystic lesion within the right pulmonary trunk extending to the right lower lobe, showing low signal intensity on T1 and high intensity on T2 images with septation (see Figures 4 and 5). Parenchymal hydatid cysts were noted in the bilateral lower lobes, with the presence of adjacent atelectasis and small pleural effusion on the left side (see Figure 6). Images of the upper abdomen confirmed the large hydatid cyst in the right liver lobe. Surgical intervention was discussed with the family and they decided against it. The patient was discharged on oral albendazole 400 mg twice daily and oral praziquantel 1800 mg twice weekly. After ten months, repeat CT and MRI showed mild regression in size of both, the right main pulmonary artery and left sided parenchymal hydatid cysts. Praziquantel was stopped and presently the patient is on therapy with oral albendazole 400 mg twice daily.

## 3. Discussion

Primary echinococcosis can be found in any organ; secondary echinococcosis may happen because of the spread of metacestodes from the primary site [1]. Involvement of the pulmonary artery with echinococcal cysts is very rare and occurs mostly due to embolization of daughter cysts from other organs like the liver or the heart [3]. The embolization mostly happens as a result of rupture of a hydatid cyst located in the right chambers of the heart or as a result of migration of daughter cysts from the liver to the pulmonary arteries [4]. In the present case, there was a history of hydatid liver disease prior to presentation and small parenchymal pulmonary cysts were also present. We hypothesized that the cysts in the lung parenchyma may have caused injury to the vessel wall through which parasites were able to cross into the lumen, although dissemination through the liver could not be excluded.

Primary infection in the lung parenchyma may remain asymptomatic for many years and most intact lung cysts are discovered incidentally on chest radiographs [1]. Symptoms from unruptured cysts may include cough, hemoptysis, or chest pain [5]. Cyst located in an artery may grow slowly and occlude it [6]. In the present case, the patient was asymptomatic for years and this agrees with the slow growth rate of cysts located in pulmonary artery which afforded enough time for collaterals to develop. Symptoms may develop when the cyst enlarges to more than 20 cm in diameter and compresses a vital structure or when it ruptures, causing anaphylactic shock [1, 7, 8]. Anaphylactic shock may cause death when the cyst material is released, due to trauma or surgery [9]. In our case, the patient complained of mild shortness of breath, which could be due to decreased pulmonary perfusion.

Diagnosis of hydatid cyst can be made using a combination of radiological imaging and serological tests [4]. Ultrasonography is the preferable method to diagnose cystic lesions. CT and MRI with heavily T2-weighted series may be the imaging of choice when the location is not accessible [10]. In our patient, the diagnosis was made using CT and MRI images. On CT, the cysts in the arterial lumen appeared as rounded intravascular masses with levels of fluid attenuation and smooth borders surrounded by normal lung tissue, with contrast enhancement at the periphery [1]. MRI images may differ, depending on the developmental phase of the cyst; it will show low signal intensity on T1-weighted images and high signal intensity on T2-weighted images [11]. Pulmonary thromboembolism and primary arterial tumors like sarcomas are the top differential diagnoses for the observed lesion. Pulmonary thromboembolism will be excluded due to the lesion cystic appearance in CT and MRI while thromboembolism will show solid lesion appearance. Arterial tumor would have a faster rate of growth and more diffuse contrast enhancement [12].

Treatment options for hydatid cysts include surgical and medical approaches. The approaches to the treatment of this rare condition are not standardized, are underreported, and should be personalized considering the location, size, and adherence of the hydatid cyst to the artery. Surgical options range from puncture and aspiration of the cyst content to partial resection of the whole organ [13]. The surgical approach is most appropriate for complicated cysts (rupture, compression of vital organs and vessels, hemorrhage, and secondary bacterial infection) [13]. Albendazole is considered the main medical treatment for hydatid liver cyst and has limited success [14].

The present case report highlights the fact that, in adults with a history of hydatid liver cyst presenting with clinical features suggestive of PE, the uncommon location of hydatid cyst in the pulmonary artery should also be considered as a possibility.
